# Distinction between rhinitis alone and rhinitis with asthma using interactomics

**DOI:** 10.1038/s41598-023-39987-6

**Published:** 2023-08-12

**Authors:** Daniel Aguilar, Nathanaël Lemonnier, Erik Melén, Mariona Bustamante, Olena Gruzieva, Stefano Guerra, Thomas Keil, Gerard H. Koppelman, Juan C. Celedón, Josep M. Antó, Jean Bousquet

**Affiliations:** 1 6AM Data Mining, Barcelona, Spain; 2 Institute for Advanced Biosciences, UGA-INSERM U1209-CNRS UMR5309, Site Santé, Allée des Alpes, La Tronche, France; 3 Department of Clinical Science and Education, Södersjukhuset, Karolinska Institutet, Stockholm, Sweden; 4 Sach’s Children and Youth Hospital, Södersjukhuset, Stockholm, Sweden; 5 ISGlobal, Barcelona Institute for Global Health, Barcelona, Spain; 6 Universitat Pompeu Fabra (UPF), Barcelona, Spain; 7 CIBER Epidemiología y Salud Pública (CIBERESP), Madrid, Spain; 8 Institute of Environmental Medicine, Karolinska Institutet, Stockholm, Sweden; 9 Centre for Occupational and Environmental Medicine, Region Stockholm, Stockholm, Sweden; 10 Asthma and Airway Disease Research Center, University of Arizona, Tucson, AZ USA; 11 Institute for Clinical Epidemiology and Biometry, University of Würzburg, Würzburg, Germany; 12 Institute of Social Medicine, Epidemiology and Health Economics, Charité-Universitätsmedizin Berlin, Berlin, Germany; 13 State Institute of Health, Bavarian Health and Food Safety Authority, Erlangen, Germany; 14 Department of Pediatric Pulmonology and Pediatric Allergology, GRIAC Research Institute, Beatrix Children’s Hospital, University Medical Center Groningen, University of Groningen, Groningen, The Netherlands; 15 Division of Pediatric Pulmonary Medicine, UPMC Children’s Hospital of Pittsburgh, University of Pittsburgh, Pittsburgh, PA USA; 16 Institute of Allergology, Charité–Universitätsmedizin Berlin, Corporate Member of Freie Universität Berlin and Humboldt-Universität zu Berlin, Berlin, Germany; 17 Fraunhofer Institute for Translational Medicine and Pharmacology ITMP, Allergology and Immunology, Berlin, Germany; 18 University Hospital of Montpellier, Montpellier, France; 19 Inserm Equipe d’Epidémiologie Respiratoire Intégrative, CESP, Villejuif, France

**Keywords:** Quality of life, Respiratory signs and symptoms, Asthma

## Abstract

The concept of “one-airway-one-disease”, coined over 20 years ago, may be an over-simplification of the links between allergic diseases. Genomic studies suggest that rhinitis alone and rhinitis with asthma are operated by distinct pathways. In this MeDALL (Mechanisms of the Development of Allergy) study, we leveraged the information of the human interactome to distinguish the molecular mechanisms associated with two phenotypes of allergic rhinitis: rhinitis alone and rhinitis in multimorbidity with asthma. We observed significant differences in the topology of the interactomes and in the pathways associated to each phenotype. In rhinitis alone, identified pathways included cell cycle, cytokine signalling, developmental biology, immune system, metabolism of proteins and signal transduction. In rhinitis and asthma multimorbidity, most pathways were related to signal transduction. The remaining few were related to cytokine signalling, immune system or developmental biology. Toll-like receptors and IL-17-mediated signalling were identified in rhinitis alone, while IL-33 was identified in rhinitis in multimorbidity. On the other hand, few pathways were associated with both phenotypes, most being associated with signal transduction pathways including estrogen-stimulated signalling. The only immune system pathway was FceRI-mediated MAPK activation. In conclusion, our findings suggest that rhinitis alone and rhinitis and asthma multimorbidity should be considered as two distinct diseases.

## Introduction

Allergic rhinitis tends to cluster with asthma (A) in multimorbidity^1^. However, clinically, two rhinitis (R) phenotypes can be identified: (i) R alone (affecting around 70–80% of patients with R) and (ii) R in multimorbidity with A (R + A), affecting 20–30%. On the other hand, the majority of patients with A have or have had rhinitis (R)^1^. Furthermore, airway remodelling, a constant feature of A, does simply not exist in R. It is also important to consider that the clinical, immunological and genetic differences between monosensitisation (to one allergen) and polysensitisation (to more than one allergen) and the link between polysensitisation and multimorbidity increase the heterogeneity of R. This suggests the existence of distinct molecular pathways in R + A and R alone^2^. In consequence, the concept of “one-airway-one-disease” (coined over 20 years ago) may be an oversimplification of the disease^1,3^.

Previous efforts to understand the links between R and R + A have focused on the atopic march sequence^4^. An alternative approach is the characterisation of the molecular mechanisms of these diseases and their interactions. A complete characterisation of cellular function can only emerge from studying how gene products interact with one another, forming a dense molecular network known as the interactome, which can be defined as the representation of all interactions (regulatory, metabolic, physical, etc.) among the gene products present at a given time within a cell. This is where the branch of systems biology, known as *interactomics,* comes into play, applying data mining and biostatistical methodologies (i) to identify molecular pathways and (ii) in general, to provide a molecular context that will facilitate the understanding of the complexity of many phenotypes. During the last decade, its analysis has provided important insights into the inner operations of the cell under different conditions including pathological ones^5,6^.

The MeDALL study, which was aimed at unravelling the complexity of allergic diseases, did show that the coexistence of eczema, R and A in the same child is more common than expected by chance alone, both in the presence and absence of IgE sensitisation. This suggests that these diseases share causal mechanisms^7^. A MeDALL in silico study suggested the existence of a multimorbidity cluster between A, eczema and R, and that type 2 signalling pathways represent a relevant multimorbidity mechanism of allergic diseases^8^. The in silico analysis of the interactome at the cellular level implied the existence of differentiated multimorbidity mechanisms between A, eczema and R at cell type level, as well as mechanisms common to distinct cell types^9^. A transcriptomics study of samples from MeDALL birth cohorts identified a signature of eight genes associated to multimorbidity for A, R and eczema^7^. In this study, genes of R alone differed from those of R + A multimorbidity without any overlap.

In this study, we used transcriptomic information obtained in MeDALL cohorts to compare the molecular mechanisms of R and R + A, assessing how the relationship between these diseases should be understood in a multimorbidity framework using an interactomics approach.

## Materials and methods

### Study design

We used the transcriptomics data from Lemonnier et al. obtained in MeDALL (Mechanisms of the Development of Allergy)^10^. The analysis comprised a cross-sectional study carried out in participants from three MeDALL cohorts using whole blood. It compared participants with single allergic disease (asthma, dermatitis or rhinitis) or with multimorbidity (A + D, A + R, D + R, or A + D + R) to those without asthma, dermatitis or rhinitis and non-allergic participants. We characterised the molecular pathways associated to R alone and R + A using an interactomics approach.

### Settings and participants

Three birth cohorts were used: BAMSE (Swedish abbreviation for Children, Allergy, Milieu, Stockholm, Epidemiology, Sweden), INMA (INfancia y Medio Ambiente, Spain) and GINIplus (German Infant Study on the Influence of Nutrition Intervention plus Air pollution and Genetics on Allergy Development, Germany)^10^.

### Datasets

Differentially expressed genes (DEGs) for R alone and for R + A were obtained from the MeDALL gene expression study^10–13^. The full dataset is supplied in Supplementary Table S1.

### The interactome

The first-degree interactomes of the DEGs for R alone and for R + A were independently generated using the IntAct database, which contains a curated collection of > 10^6^ experimentally determined protein–protein interactions in human cells^14^. Data was downloaded via the IntAct web-based tool at the European Bioinformatics Institute (https://www.ebi.ac.uk/intact/). Ensembl Gene IDs were used instead of HGNC names to avoid ambiguities. Self-interactions and expanded interactions were discarded. Interactomes are supplied in Supplementary Table S2. Network density was calculated as the number of edges with respect to the maximal possible number of edges. Random distributions were used to test the degree of interconnectedness of the interactomes. They were generated by random sampling of gene sets (of the same size) of each interactome over 10,000 iterations.

### Functional annotation

The interactomes were functionally annotated using the DAVID web-based tool^15^, with the Reactome database as the source of functional information^16^ and the default gene background. Functional pathways were considered significant with *FDR* < 0.05. In order to simplify the functional annotation and remove redundancy, pathways associated to diseased or defective cellular processes were removed and we only considered pathways in the intermediate levels (levels 3 and 4) of the Reactome hierarchy. Furthermore, we assigned each pathway to a generic functional family (“Signal Transduction”, “Cell Cycle”, etc.) in order to help interpreting the results. We did this by (1) clustering all the pathways according to their Szymkiewicz-Simpson overlap^17^; (2) identifying the best partition of using the Pearson’s Gamma method^18^ implemented in the *fpc* R package; and (3) asigning each cluster in the best partition to a generic pathway (i.e. a Reactome pathway with > 800 genes) by means of a Fisher’s Exact Test^19^. Full functional annotation is available in Supplementary Table S3.

### Software

All data mining and statistical analysis were carried out using the R programming language^20^.

## Results

### Demographic characteristics of the participants

Among the 786 participants included in the analysis, 54.8% had no allergic disease. Among those with an allergic disease, 45% had asthma (61% with multimorbidity), 42% dermatitis (48%) and 51% rhinitis (49%). Asthma was more common in BAMSE (63%), dermatitis in INMA (74%) and rhinitis in GINIplus (79%). Fifty-five percent of the participants had no allergic disease (BAMSE 39%, GINIplus 67%, INMA 54%) (Table 1).

**Table 1:** Characteristics of the population study.

	BAMSE	GINIplus	INMA	Total
Individual samples included	270	346	208	824
Samples with process fail^a^	1	1	1	3
Samples with quality fail^b^	7	11	1	19
Outliers detected^c^	6	5	5	16
Included in the analysis	256	329	201	786
Male (n, %^d^)	143 (55.9)	160 (48.6)	99 (49.2)	402 (51.1)
Female (n, %^d^)	113 (44.1)	169 (51.4)	102 (50.8)	384 (48.9)
Controls (n, %^d^)	100 (39.1)	222 (67.5)	109 (54.2)	431 (54.8)
Any allergic disease (n, %^d^)	156 (60.9)	107 (32.5)	92 (45.8)	355 (45.2)
A alone (n)	32	15	18	65
D alone (n)	22	6	48	76
R alone (n)	23	64	4	91
A + D (n)	17	1	16	34
A + R (n)	33	15	2	50
D + R (n)	13	4	4	21
A + D + R (n)	16	2	0	18
Asthma multimorbidity (n, %^e^)	66 (42.3)	18 (16.8)	18 (19.6)	102 (28.7)
Dermatitis multimorbidity (n, %^e^)	46 (29.5)	7 (6.5)	20 (21.7)	73 (20.6)
Rhinitis multimorbidity (n, %^e^)	62 (39.7)	21 (19.6)	6 (6.5)	89 (25.1)
Any multimorbidity (n, %^e^)	79 (50.6)	22 (20.6)	22 (23.9)	123 (34.6)
Any single disease (n, %^e^)	77 (49.4)	85 (79.4)	70 (76.1)	232 (65.4)
Age (years ± sd)	16.7 ± 0.4	15.0 ± 0.2	4.2 ± 0.3	12.8 ± 5.1

Demographics. A: asthma, D: dermatitis, R: rhinitis.

^a^Process fail: samples failed extraction, sample preparation or hybridisation/scan process, no CEL file available.

^b^Quality fail: samples not complying with minimal RIN or Genechip metrics thresholds.

^c^Outliers selection based on PCA as described in supplementary methods in Lemonnier et al.^10^.

^d^% of total.

^e^% of cases.

### Topological analysis of the interactomes

**Figure 1:**
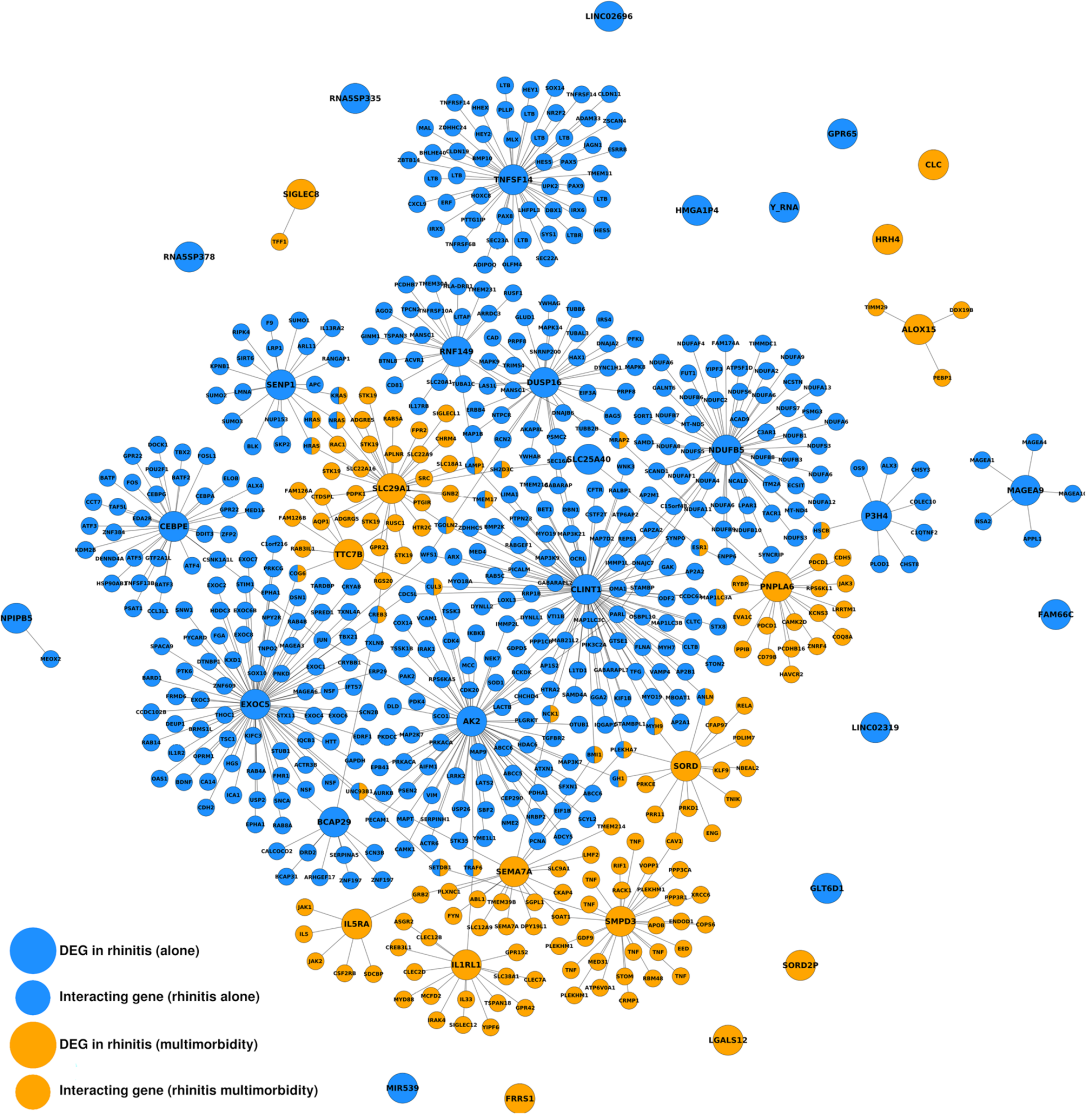
Interactomes of rhinitis alone and rhinitis associated with multimorbidity. DEGs: differentially expressed genes. For clarity, only genes with HGNC symbol are shown.

We generated interactomes for R alone and R + A, which can be seen as snapshots of the cellular mechanisms behind these conditions (Fig. 1). The interactome of R alone consisted of 464 genes connected by 466 edges. The interactome of R + A consisted of 130 genes connected by 149 edges. The interactome of R alone is 2.18 times denser than random expectation, which is statistically significant (*z*-test; *P* = 1.09·10^–11^). Similarly, the interactome of R + A is 6.22 times denser than random expectation, which is also statistically significant (*z*-test; *P* = 3.42·10^–50^). There were no DEGs common to R alone and R + A in the MeDALL study, but we identified 25 genes common to both interactomes, which implied a degree of interconnectedness significantly larger than random expectation (*z*-test; *P* = 2.52·10^–22^).

### Functional annotation

**Figure 2:**
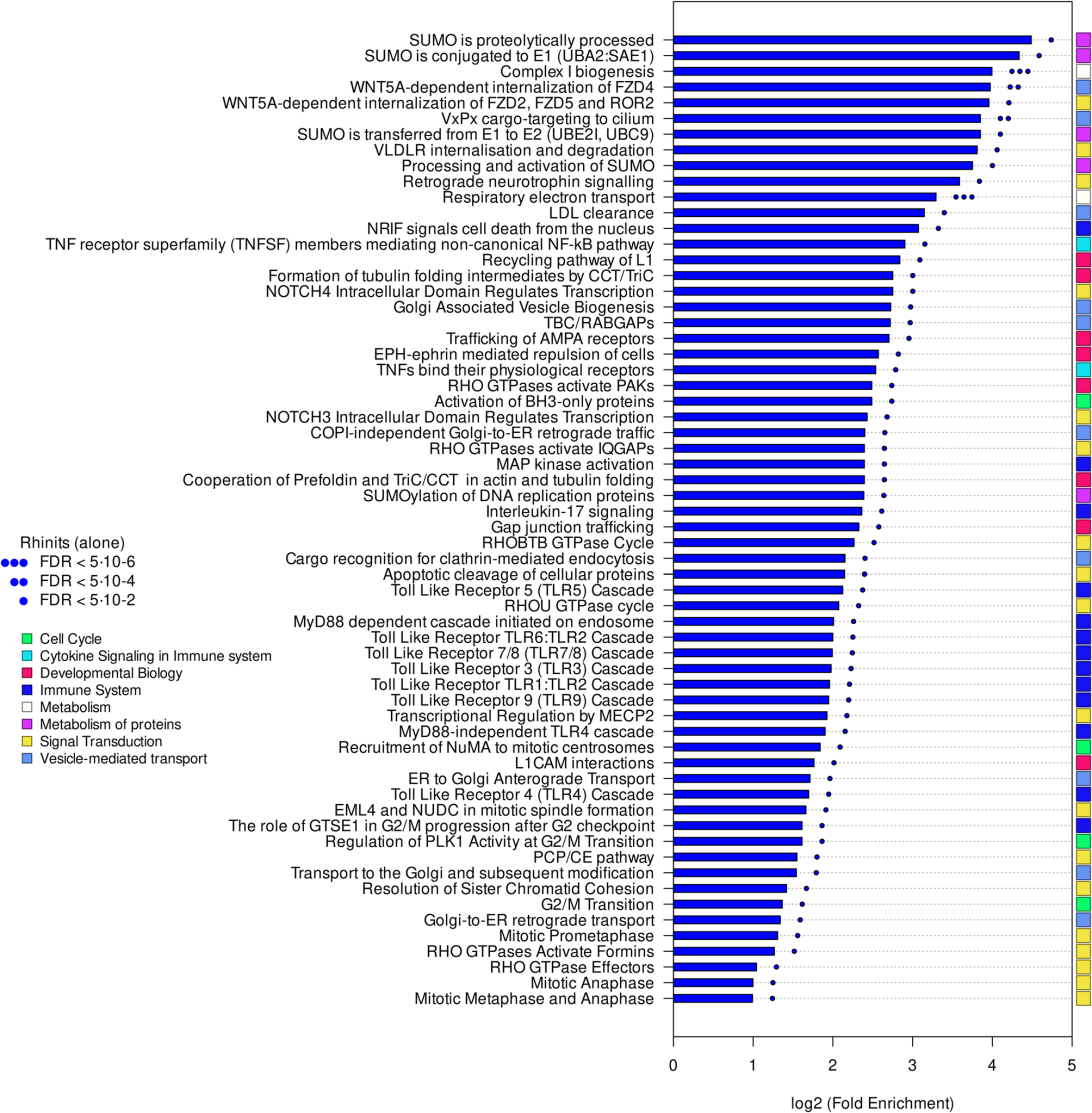
Pathways unique to rhinitis alone. Pathways were classified in broad functional families (coloured rectangles).

**Figure 3:**
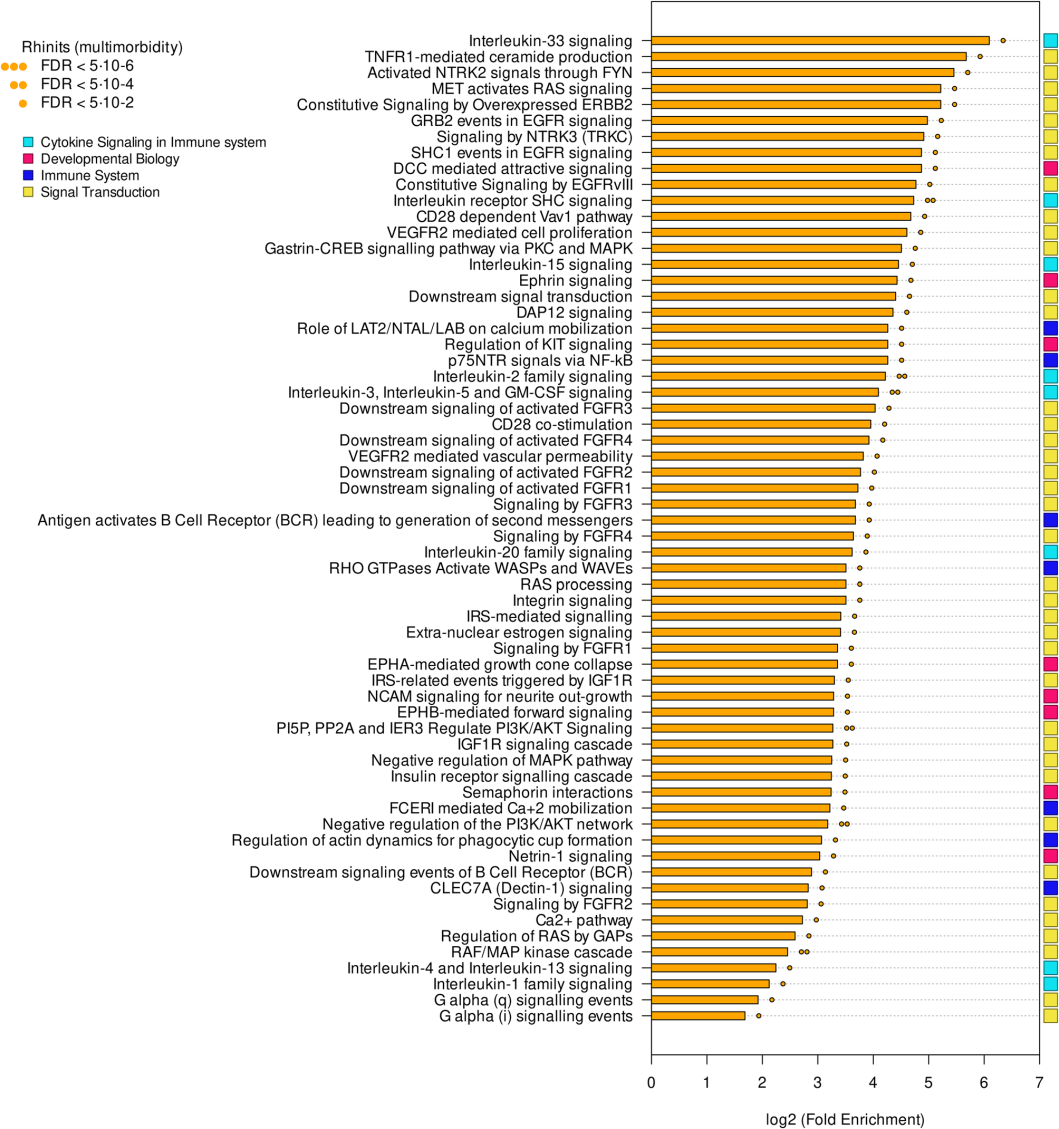
Pathways unique to rhinitis in multimorbidity. Pathways were classified in broad functional families (coloured rectangles).

Functional annotation revealed marked differences in the molecular pathways of both phenotypes. Pathways specific to R alone (Fig. 2; in table form in Supplementary Table S4) involved a number of Toll-like receptor (TLR), IL-17 and MyD88 (myeloid differentiation primary response gene 88) signalling cascades, as well as WNT5A-dependent signalling, RHO GTPase activity and the small ubiquitin-related modifier (SUMO) pathways. In contrast, pathways associated to R + A (Fig. 3; in table form in Supplementary Table S4) were much richer in signal-transduction-related processes such as IL-mediated and fibroblast growth factor receptors (FGFRs)-mediated signalling. IL-33 particularly stood out with a ~ 68-fold enrichment.

**Figure 4:**
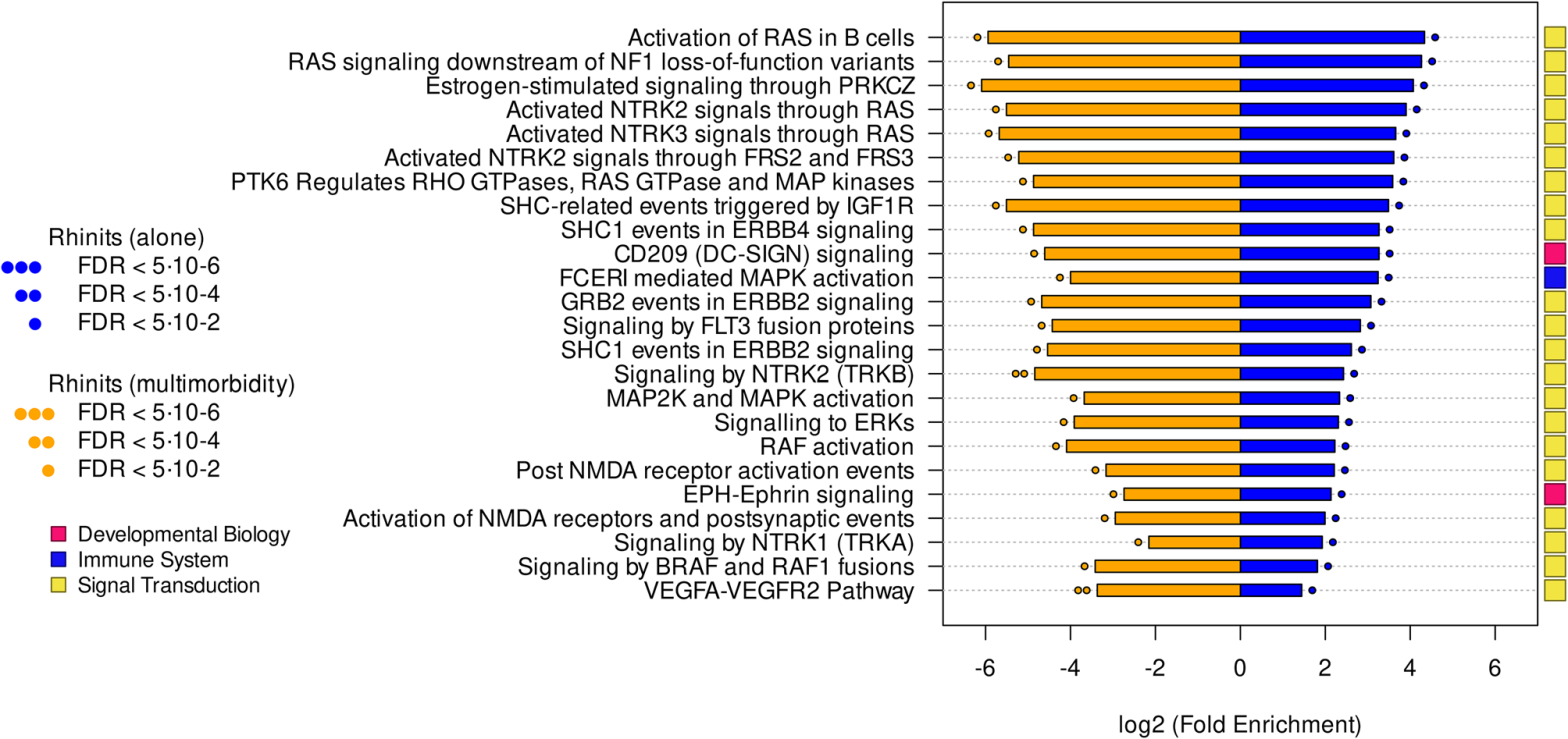
Pathways common to rhinitis alone and rhinitis in multimorbidity. Pathways were classified in broad functional families (coloured rectangles).

The pathways common to both R phenotypes are shown in Fig. 4 (in table form in Supplementary Table S4). The pathways with largest fold enrichment (both in R alone and R + A) are estrogen-stimulated signalling through PRKCZ and RAS-mediated signalling.

## Discussion

Using topological and functional analysis, we identified a core of common mechanisms between the two phenotypes of R, but also found significant differences between both phenotypes. Densely interconnected groups of genes within the interactome are known to be contributors to the same pathological phenotypes^21^. The high level of connectivity that we observed within the interactome of each phenotype, together with the lack of common DEGs, suggests that R alone and R + A are largely mechanistically different diseases, affecting different molecular pathways.

TLRs stand out as strong drivers of R alone. TLRs are type I transmembrane receptors employed by the innate immune system^22^. Variation in the TLR genes has been associated with R in several candidate gene studies. A significant excess of rare variants in R patients was found in *TLR1*, *TLR5*, *TLR7*, *TLR9* and *TLR10* but not in *TLR8*^23^. Children carrying a minor rs1927911 (*TLR4*) allele may be at a higher R risk^24^. In turn, TLR is strongly associated to MyD88 pathways, mediating in innate lymphoid cells type 2 (ILC2) activation and eosinophilic airway inflammation^25,26^. *IL-17* was also identified, as were SUMO pathways, known to regulate many cellular processes including signal transduction and immune responses^27,28^. Furthermore, it is known that SUMOylation plays a critical role in the expression of TSLP in airway epithelial cells. Inhibition of SUMOylation attenuates house dust mite-induced epithelial barrier dysfunction^29^.

On the other hand, there are a number of mechanisms—such as Nf-kB-mediated signalling and *IL-1* and *IL-33* activity—that seem to be driving R + A multimorbidity. *IL-33* and *IL1RL1* are among the most highly replicated susceptibility loci for A^30^, and IL-33 has a known role in infection-mediated A susceptibility^31^. There is an increase in FGFR (fibroblast growth factor receptor) signalling. The FGF/FGFR signalling system regulates a variety of biological processes, including embryogenesis, angiogenesis, wound repair and lung development^32^. It may be relevant in A remodelling. Interleukin-33 (IL-33) which belongs to the interleukin-1 (IL-1) family is an alarmin cytokine with critical roles in tissue homeostasis, pathogenic infection, inflammation, allergy and type 2 immunity. IL-33 transmits signals through its receptor IL-33R (also called ST2) which is expressed on the surface of T helper 2 (Th2) cells and group 2 innate lymphoid cells (ILC2s), thus inducing transcription of Th2-associated cytokine genes and host defense against pathogens^33^. IL33 and IL1RL1/ST2 are among the most highly replicated susceptibility loci for asthma^34,35^. However, IL-33 is not associated with rhinitis alone^36^.

The exposure of the airway epithelium to external stimuli such as allergens, microbes, and air pollution triggers the release of the alarmin cytokines IL-25, IL-33 and thymic stromal lymphopoietin (TSLP). IL-25, IL-33 and TSLP interact with their ligands, IL-17RA, IL1RL1 and TSLPR, expressed by cells including dendritic cells, ILC2 cells, endothelial cells, and fibroblasts. Alarmins play key roles in driving type 2-high, and to a lesser extent type 2-low responses, in asthma^1,3,37^. Future analysis could exploit tissue-specific transcriptomic data to highlight the differences between the epithelium of upper and lower airways^38,39^.

Finally, some signal transduction pathways common to R alone and R + A have an impact on the IgE-mediated immune response. They include activation of *RAS* on B cells, CD209 signalling^40^, MAPK Kinase^41^, FceRI MAPK kinase activation^42^, ERK activation^43^, Raf kinases^44^ or VEGFA^45^.

## Limitations of the study

One major problem is that there are no data on A without rhinitis. However, this is not the first study in which either the A population is too low or there is no signal for A alone^10^. This is the case for the present study and we were unable to include the A alone group. It is likely that A is almost always associated with R in children.

Incompleteness and spurious interactions have for a long time been limitations in studies that make use of data from the human interactome. However, authors have argued that data noise does not limit a successful application of the interactome to the investigation of disease mechanisms^46,47^. Also, the human interactome is known to be biased towards certain genes of interest (a category that includes many disease-associated genes)^48^. However, non-biased interactomes have a much lower coverage, which makes them unsuitable for some topology-based studies^49^. Lastly, time-dependent and location-dependent interaction patterns are not captured in our study, which only considers an interactome static in time.

## Impact of the study

Clinical data, epidemiologic studies^50^, mHealth-based studies^51^ and genomic approaches^7^ all support the existence of two distinct diseases: R alone and R with A multimorbidity. This study helps to better understand the differences between R and R + A and to refine the ARIA-MeDALL hypothesis on allergic diseases^52^. It also highlights the importance of IL-17^53^, IL-33^54^ and their interactions to understand the allergic multimorbidity.

## Conclusions

The interactomes of R alone and R + A showed topological characteristics that suggest that the cellular mechanisms involved are different for each phenotype. We identified mechanisms specific to R alone (TLR and MyD88 signalling cascades, SUMO pathways) and mechanisms specific to R + A (IL-33-mediated signalling, FGFR-mediated signalling).

### Supplementary Information

Supplementary Information.

## Data Availability

The datasets supporting this work are available as supplementary materials. Transcriptomics data was downloaded from Lemonnier et al.^9^.
